# Supplementary material for: Mechanisms governing the pioneering and redistribution capabilities of the non-classical pioneer PU.1
Source: Nat Commun. 2020 Jan 21;11:402. doi: 10.1038/s41467-019-13960-2 (PMC6972792; doi:10.1038/s41467-019-13960-2)
Supplement: Supplementary file 7 — Source data [file 41467_2019_13960_MOESM7_ESM.zip › Source_Data/FigureS3/FigureS3J_MotifScanOutput/homerResults/motif37.similar.html]

motif37

## Information for motif37

G
T
C
A
A
T
G
C
C
G
T
A
C
T
A
G
C
T
A
G
T
A
C
G
A
G
T
C
A
G
C
T
A
C
T
G
  
Reverse Opposite:  

T
G
A
C
T
C
G
A
A
C
T
G
A
T
G
C
G
A
T
C
A
G
T
C
C
G
A
T
A
T
C
G
C
A
G
T
  

|  |  |
| --- | --- |
| p-value: | 1e-34 |
| log p-value: | -8.024e+01 |
| Information Content per bp: | 1.643 |
| Number of Target Sequences with motif | 3580.0 |
| Percentage of Target Sequences with motif | 27.13% |
| Number of Background Sequences with motif | 7940.7 |
| Percentage of Background Sequences with motif | 21.75% |
| Average Position of motif in Targets | 500.5 +/- 490.4bp |
| Average Position of motif in Background | 318.7 +/- 206.7bp |
| Strand Bias (log2 ratio + to - strand density) | -0.0 |
| Multiplicity (# of sites on avg that occur together) | 1.32 |
| Motif File: | file (matrix) reverse opposite |

### Similar de novo motifs found

|  |  |  |  |  |  |  |  |
| --- | --- | --- | --- | --- | --- | --- | --- |
| Rank | Match Score | Redundant Motif | P-value | log P-value | % of Targets | % of Background | Motif file |
| 1 | 0.870 | T A G C C A T G T C A G A T G C G A T C A G T C G C A T T A C G | 1e-26 | -61.035441 | 30.61% | 25.72% | motif file (matrix) |
| 2 | 0.821 | T C G A A C T G A T G C G A T C A G T C C G A T A T C G A T G C G T A C T A C G | 1e-24 | -55.790070 | 24.00% | 19.72% | motif file (matrix) |
| 3 | 0.870 | C T A G A C T G A G T C A G T C A G T C A C G T A C T G | 1e-20 | -46.865685 | 20.73% | 17.04% | motif file (matrix) |
